# Characterization of lactobacilli strains isolated from baby’s feces for their potential immunobiotic application

**Published:** 2019-10

**Authors:** Imad Al Kassaa, Samah Mechemchani, Mazen Zaylaa, Mohamad Bachar Ismail, Khaled El Omari, Fouad Dabboussi, Monzer Hamze

**Affiliations:** 1 Department of Health and Environment Microbiology Laboratory, Doctoral School of Sciences and Technology and Faculty of Public Health, Lebanese University, Tripoli, Lebanon; 2 Quality Control Center Laboratories at the Chamber of Commerce, Industry Agriculture of Tripoli and North Lebanon, Tripoli, Lebanon

**Keywords:** Lactobacilli, Immunomodulation, Immunobiotic, Probiotic, Inflammatory bowel disease

## Abstract

**Background and Objectives:**

Several LAB species were evaluated and characterized for potential probiotic use. Besides the antimicrobial activity, probiotics showed recently a capacity to prevent and to alleviate inflammatory and chronic diseases. Immunomodulation effect is one of the modes of actions of such probiotics, called immunobiotics, which can be used in several chronic diseases such as Inflammatory Bowel Diseases (IBD). The aim of this study was to isolate, identify and characterize lactobacilli strains from healthy baby’s feces in order to select some strains with potential immunobiotic application especially strains which can stimulate anti-inflammatory responses.

**Materials and Methods:**

Forty-two LAB strains were isolated and identified by the MALDI-TOF / MS technique. In addition, strains were subjected to several assessments such as antimicrobial activity, the capacity to form biofilm in polystyrene microplate and immunomodulation activity in a PBMC model.

**Results:**

Results showed that the majority of strains (90.4%) were identified as *Lactobacillus*. However, among these, only 39.4% of lactobacilli strains were not identified at the species level. All isolated lactobacilli strains showed an anti-inflammatory effect. Moreover, 7 strains were considered as good probiotic candidates based on their characteristics such as their antibacterial activities, formation of the strongest biofilm and their ability to stimulate an anti-inflammatory response in PBMCs model.

**Conclusion:**

Two strains (*Lactobacillus* spp S14 and *Lactobacillus* spp S49) which showed the best immunobiotic characteristics, could be selected and evaluated more deeply *in vivo* model as well as in human clinical study to ensure their effectiveness in inflammatory diseases such as IBD.

## INTRODUCTION

More than 100 trillion symbiotic microorganisms live on and within human beings and play a crucial role in human health. The human microbiota, especially the gut microbiota, has even been considered to be an “essential organ” (1) carrying approximately 150 times more genes that are found in the entire human genome (2). This large microbial community performs many biological and metabolic functions and provides many beneficial effects to the host. The role of a healthy intestinal microbiota is to maintain homeostasis, then, the ecosystem falls into a balance between proinflammatory and anti-inflammatory responses. However, this microbiota may declines with age due to malnutrition, antibiotic therapy and other extrinsic factors, which causes a state of dysbiosis (3). Like the concept of the pathogenicity of a single microbial taxon, dysbiosis of a microbial community can be difficult to define but could be considered as a perturbation that departs from an otherwise balanced ecology (4) to prolong, exacerbate, or induce a detrimental health effect. Many chronic diseases such as obesity, Inflammatory Bowel Disease (IBD), diabetes mellitus, metabolic syndrome, atherosclerosis, Alcoholic Liver Disease (ALD), Nonalcoholic Fatty Liver Disease (NAFLD), cirrhosis, and hepatocellular carcinoma have been associated with the human gut microbiota dysbiosis (5, 6). Hence the search for a treatment to restore intestinal homeostasis in order to prevent dysbiosis, the hidden disease of our decade, becomes crucial. In this context, several studies showed the beneficial effect of some micro-organisms which colonize human and animal mucosa (7). An important example of such micro-organisms is *Lactobacillus*. They belong to the group of lactic acid bacteria (LAB) and are often found in fermented food and in the digestive tract of humans and animals (8). Lactobacilli strains can fight pathogens via several mechanisms. Strong colonization and formation of biofilm, secretion of bacteriocins as well as other antimicrobial substances and enhancement of local and systemic immunity are the main anti-pathogenic mechanisms of these bacteria (9–13). These beneficial strains are called probiotic strains which have had several definitions for more than a hundred of years, and which were finally defined as “living or dead microorganisms that exert a beneficial effect on the immune function, intestinal microbiology, or physiology of the host when ingested in sufficient amounts” (14). The use of probiotics as preventive agents to enhance immunity and reduce infection is widely common (15). The beneficial effect of probiotics is simply to restore the function of normal microbiota (15). The aim of this study is to characterize probiotic properties of LAB strains isolated from healthy baby’s feces. The antimicrobial activity, biofilm formation, and immunomodulation effect of each isolated strain were evaluated.

## MATERIALS AND METHODS

### Sample collection and LAB isolation

Stool samples were collected from healthy babies aged less than 4 months. Fecal samples were collected either from the diaper or by rectal swabbing (n=72). After serial dilution, samples were cultured on the Man, Rogosa and Sharpe agar medium (MRS, Bio-Rad, France). The culture was incubated for 24 or 48 hours at 35°C in anaerobic conditions by using anaerobic bags (Biomérieux, France).

In order to avoid the growth of unwanted species, in particular enterococci, the subculture of suspected colonies was carried out using a selective medium of the following composition: Columbia agar (Bio-Rad, France) supplemented with glucose, lactulose, cysteine HCL, riboflavin (Sigma-Aldrich, Germany), propionic acid and Mupirocin (GlaxoSmithKline, France) as an antibiotic. This medium is specific for the growth of lactobacilli and other LAB strains, and subsequently inhibits the growth of enterococci and yeasts (16). All mothers accepted to participate in this study and signed informed consent prior sample collection.

### Identification of isolated LAB strains

The identification of LAB strains was carried out at two levels, preliminary identification and molecular identification. The preliminary identification is based on Gram staining and the catalase test. Then LAB strains were identified by VITEK® MS (BioMérieux, France) which is an automated microbial identification system using mass spectrometry using Matrix-Assisted Laser Desorption Deionization Time of Flight (MALDI-TOF) technology.

### Inhibition of pathogenic microorganisms by intestinal LAB strains: Microplate method

To prepare the supernatants of the isolated strains, 1 MacFerland (McF)≈ 3×10^8^ Colony Forming Unit (CFU)/ml) of each strain was prepared in a medium containing sterile BHI broth (Bio-Rad, France) and incubated for 48 hours at 35°C in anaerobic conditions. The supernatant obtained after centrifugation of the culture (4000g/15min) was filtered by using 0.45 μm filter (Millipore, USA), exposed to heat (90°C/15min) in the water bath. Then 50 μl of BHI broth contain 10^3^ CFU/ml of each pathogen (*L. monocytogenes* ATCC^®^ 19115™ (P1) and *E. coli* ATCC^®^ 8739™(P2)) was added with 50 μl of the supernatant of each strain in 96-well microplate. The microplates were incubated aerobically at 35°C. The Optical Density (OD) of different well was measured by ELISA reader (Biotek, USA) at 630 nm every 2 hours for 18 hours. BHI was used as negative control and *E. faecium* CMUL20-2 (17) was used as positive control for *L. monocytogenes*.

### Capacity of the isolated strains to form biofilms

The capacity of forming a biofilm by the isolated strains was evaluated using Tissue Culture Plate method (TCP) as described before with slight modification (18): 1 McF (≈ 3×10^8^ CFU/ml) of each strain was prepared in a medium containing sterile MRS broth and incubated for 24 hours at 35°C in anaerobic conditions, then 200 μl of the bacterial culture (1 McF) was added in sterile 12 well flat-bottomed polystyrene microplate containing 2 ml of MRS broth. The plate was incubated at 35°C for 24 hours under CO_2_ condition. The content of the wells was poured off and washed 3 times with 2 ml of sterile distilled water. The bacteria adhering to the wells were fixed with 2 ml of methanol for 15 min. Then the wells were washed with sterile distilled water, followed by staining with 2 ml of 1% crystal violet solution for 5 min. Excess stain was removed by washing and air dried. The dye bound to the wells was extracted with 2 ml of 33% glacial acetic acid for 10 min. Then 1.5 ml of acetic acid was removed from each well, and the optical density was measured at 595 nm using a spectrophotometer (Thermo Fisher Scientific, USA). Each strain was tested three times (3 independent wells). Sterile MRS was used as negative control; commercial probiotic strain (*Lactobacillus paracasei*, Bion3-France) was used as positive control.

In order to evaluate the capacity of forming a biofilm by the isolated lactic strains, the following referred formulas have been used: If OD_C_ < OD_S_ < 2×OD_C_: The biofilm is considered as weak biofilm, if 2×OD_C_ < OD_S_ < 4×OD_C_: the biofilm is considered as moderate biofilm, if 4×OD_C_ < OD_S_: the biofilm is considered as strong biofilm, with “S”: lactic strain tested and C: Negative control” (19). On the other hand, in order to compare the capacity of forming a biofilm by isolated strains compared with the probiotic strain, the following formula was used: (OD_S_ / OD_Pb_) ×100 with “S: Lactic strain tested” and “Pb”: Probiotic strain”.

### *In vitro* immunomodulatory properties of the isolated strains

Peripheral Blood Mononuclear Cells (PBMCs) were collected by authorized staff from human blood obtained from five healthy informed donors upon approved agreement (signed consents), as previously described by Foligne et al. (20). All healthy donors signed an informed consent about this study prior blood collection. Breify, the blood was placed on a Ficoll gradient (Pharmacia, Stockholm, Sweden) and PBMCs were recovered at the interface after centrifugation, washed with PBS and adjusted to 2×10^6^ cells/ml in Roswell Park Memorial Institute (RPMI) 1640 (Gibco, Scotland) supplemented with 10% heat-inactivated fetal calf serum (Gibco, Scotland), 1 mM glutamine and 150 μg/ml gentamicin. Cells were counted and adjusted by using a hemocytometer slide. PBMCs were plated in 24-well cell culture plates and stimulated with the isolated strains at a bacteria/cell ratio of 10:1 (20 μl of a thawed bacterial suspension at 10^9^ CFU/ml in well). After 24 hours of stimulation at 37°C under 5% CO_2_, supernatants were collected, clarified by centrifugation and stored at −20°C for cytokine assay. Interleukin (IL)-10 and IL-12p70 were measured by ELISA using ELISA MAXTM Deluxe kits (BioLegend, San Diego, USA) according to the manufacturer’s instructions. Phosphate Buffer Salt (PBS) containing 20% glycerol was used as negative control, *Bifidobacterium longum* CMUL CXL 001 (*B. longum* CMUL CXL 001) was used as positive control for IL-10 (21), and *Enterococcus munditii* IAK (*E. munditii* IAK from “Collection Microbiologique de l’UniversitéLibanaise” (CMUL) bank) was used as positive control for IL-12p70.

## RESULTS

### Isolation and identification of LAB strains

**Fig. 1:**
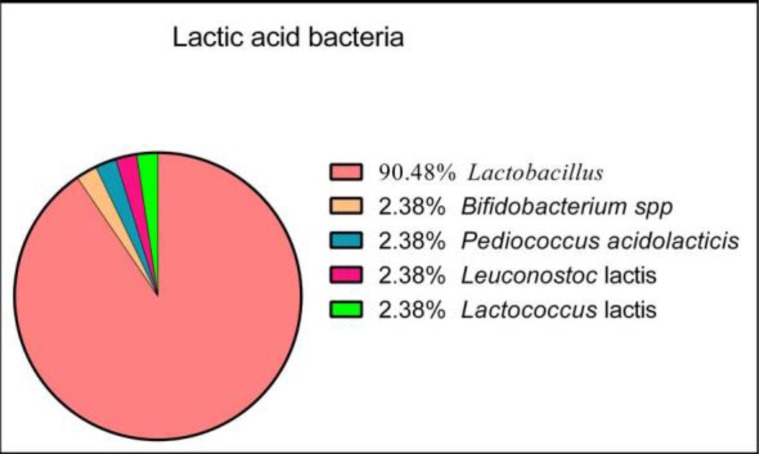
Percentage of different isolated LAB

**Fig. 2:**
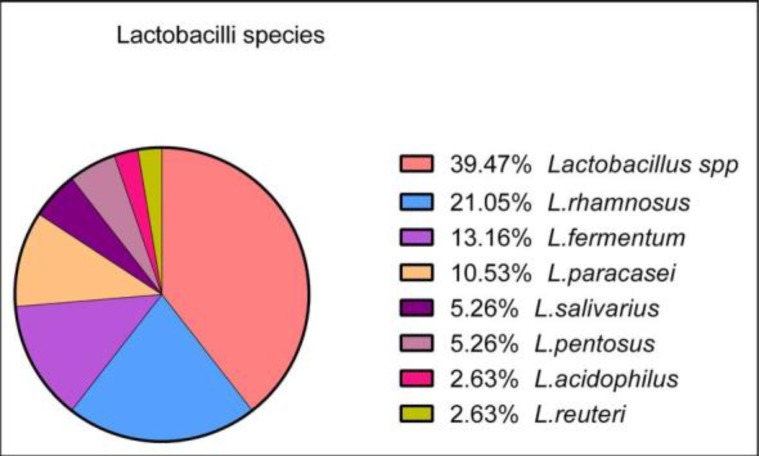
Percentage of different species of isolated lactobacilli species

Seventy-two feces samples were collected. From these, forty-two samples were positive for the presence of LAB strains. Subcultures performed on blood agar base (Bio-Rad, France) showed that the colonies were not hemolytic. Based on the identification of colonies by the MALDI-TOF VITEK^®^ MS technology, our results showed that the 42 strains isolated belong to the following genera and species: 38 *Lactobacillus*, 1 *Bifidobacterium* spp, 1 *Pediococcus acidolactici*, 1 *Leuconostoc lactis* and 1 *Lactococcus lactis* (Fig. 1). Among *Lactobacillus*, the following species were identified: 15 *Lactobacillus* spp., 8 *L. rhamnosus*, 5 *L. fermentum*, 4 *L. paracasei*, 2 *L. salivarius*, 2 *L. pentosus*, 1 *L. acidophilus*, 1 *L. reuteri* (Fig. 2).

### Biofilm formation by LAB strains

The 42 isolated LAB strains were evaluated for their ability to form biofilms on hydrophobic supports using 12-well microplates. Results show that: Biofilm production for 21 strains was strong according to this formula: 4×OD_C_ < O.D.S., biofilm production for 15 strains was moderate according to this formula: 2×OD_C_ <OD_S_ < 4×OD_C_., and biofilm production for 6 strains was weak according to this formula OD_C_ <OD_S_ < 2×OD_C_.

**Fig. 3:**
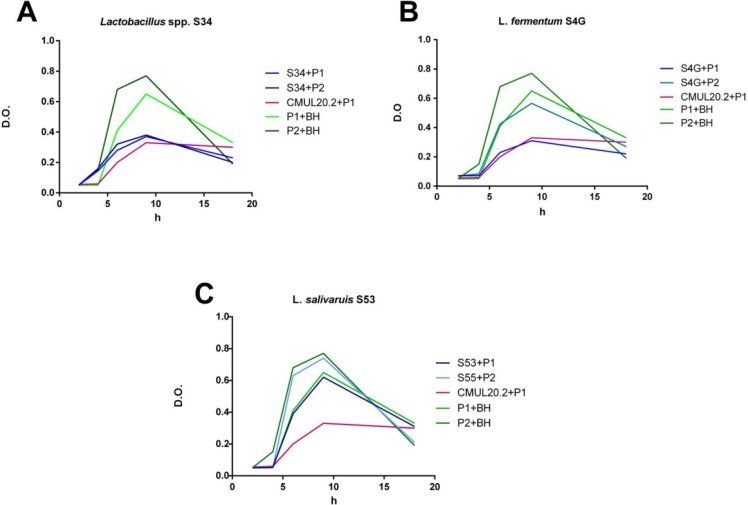
Percentage of biofilm formation of LAB strains. OD: Optical Density

The different biofilms formed by these strains were compared with the biofilm formed by a control strain consisting of a marketed probiotic (*Lactobacillus paracasei*, Bion3, France). Fig. 3 shows the different percentages of biofilm formation capacity of each strain calculated according to the formula (OD_S_ / OD_Pb_) ×100. Results showed that some of our isolated strains possess even a stronger capacity to form a biofilm than the control strain, e. g. *Lactobacillus* spp. S49 (209.40%). On the other hand, there were strains that showed a very low capacity to form biofilm such as *L. salivarius* S61 (21.90%).

### Detection of antimicrobial activity

The 42 LAB isolated strains were evaluated for their antibacterial effect; all strains were compared with a control strain producing bacteriocin, *E. faecium* CMUL20-2. From all the examined isolated strains: three strains (S4P, S4G, S19) showed activity against *L. monocytogenes* “P1”, three strains (S9P, S9G and S33) showed activity against *E. coli* “P2”, and sixteen strains (S8P, S8G, S10, S14, S18, S20G, S20P, S21, S23, S25, S30, S34, S36, S49, S55, S67) showed activity against the 2 pathogens. The other strains were inactive or had a very low activity against these pathogens.

**Fig. 4:**
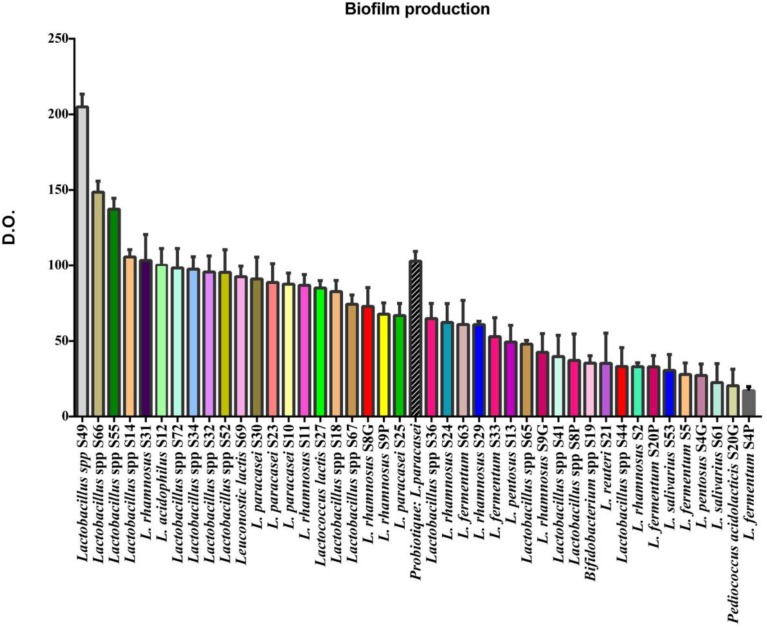
Growth curves of pathogens in the presence and absence of 3 LAB supernatant *Lactobacillus* spp. S34, *L. fermuntum* S4G and *L. salivarius* S53. The data represent the means of three independent experiments. P1: *L. monocytogenes*; P2: *E. coli*, CMUL20.2: *E. faecium* CMUL20.2, BHI: negative control.

Fig. 4 is an example of 3 strains, the first strain shows an activity against the 2 indicator strains “P1” and “P2” (A), the second strain shows an activity against only “P1” (B) and the third strain has no activity against the 2 indicator strains (C). Fig. 4 (A) shows the growth curves of indicator strains “P1” and “P2” in the absence of a supernatant of *Lactobacillus* spp. S34 strain (GCP1, GCP2), as well as in the presence of this supernatant (GC (P1/P2) S34). The supernatant of *Lactobacillus* spp. S34 strain was active against both pathogen “P1” and “P2”. This effect was observed by the large gap between GCP1 and GCP2 on the one hand and GCP1S34 and GCP2S34 respectively on the other. Compared to the control strain, GCP1CMUL20-2 and GCP1S34 are almost superposed. Fig. 4 (B) shows GCP1 and GCP2, and CCP1S4G and CCP2S4G, in the absence and presence of a supernatant of *L. fermentum* S4G strain respectively. The supernatant of *L. fermentum* S4G strain was active against the pathogen P1 only since there is a lag between GCP1 and GCP1S4G. In addition, GCP1S4G and GCP1CMUL20-2 are very close. However, a slight delay was detected between GCP2 and GCP2S4G. Fig. 4 (C) shows GCP1 and GCP2, and GCP1S53 and GCP2S53, in the absence and presence of a supernatant of *L. salivarius* S53 strain respectively. The supernatant of *L. salivarius* S53 strain appears to be inactive since there is no significant difference between GCP1 and GCP2 on the one hand and GC(P1/P2) S53 on the other hand.

### Immunomodulatory effect of LAB strains

**Fig. 5:**
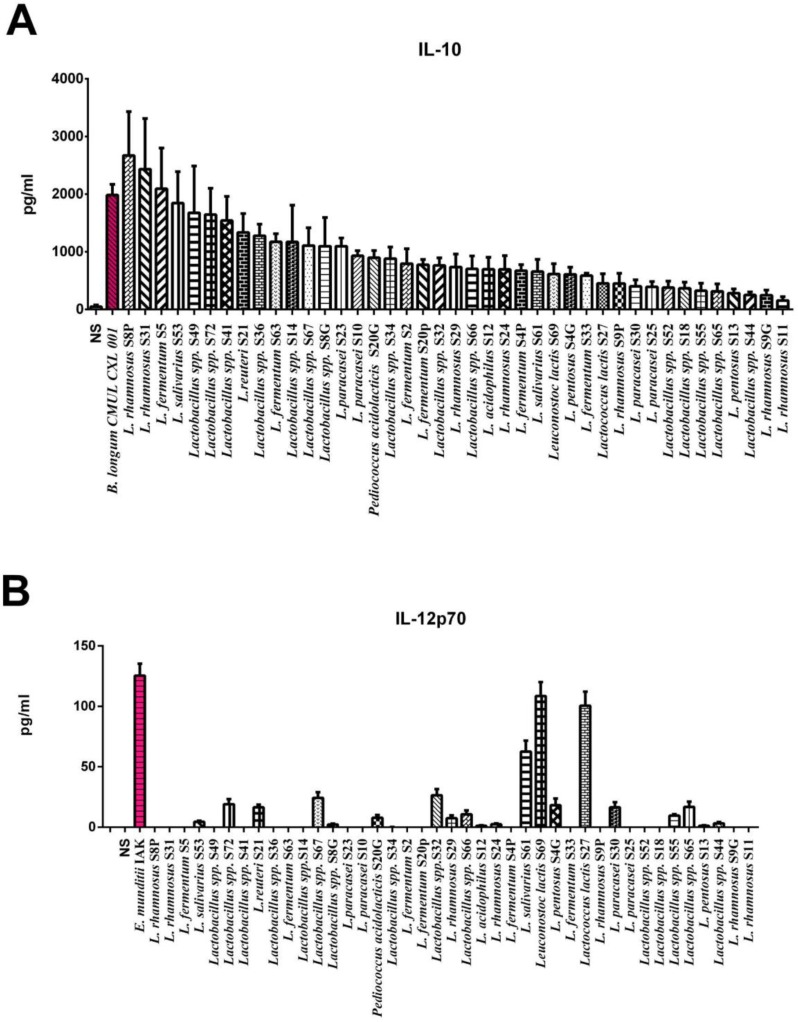
*In vitro* immunomodulatory profiles of the isolated LAB strains. Cytokine production was evaluated in the supernatants of PBMCs (n=5) different donors stimulated for 24 h by the tested strains and two control strains (*B. longum* CMUL CXL 001 and *E. munditii* IAK), in comparison to non-treated cells (NTS). Results indicate levels of (A) IL-10 and (B) IL-12p70. Data represent means ± SEM of 5 independent donors.^*^refers to the comparison of bacteria-stimulated PBMCs versus untreated cells; ^*^p < 0.05, ^***^p < 0.001.

To determine the immunomodulatory effect of the selected intestinal strains, PBMCs were stimulated and cytokine production was assessed by ELISA. Two control strains were used; *B. longum* CMUL CXL 001 and *E. munditii* IAK that respectively exhibit anti-inflammatory effect (1985.29 pg/ml of IL-10) and pro-Th1 effect (125.46 pg/ml of IL-12). As shown in Fig. 5, all intestinal strains were able to induce the secretion of the anti-inflammatory cytokine IL-10, with values ranging between 157.87 and 2670.54 pg/ml. On the other hand, most of the strains induced very low levels of pro-Th1 IL-12, except *Leuconostoc lactis* S69, *Lactococcus lactis* S27 and *L. salivarius* S61 that induce IL-12 with respective values of 108.53; 100.53 and 62.53 pg/ml, albeit below the level of the reference strain *E. munditii* IAK. *L. rhamnosus* S8P, *L. rhamnosus* S31 and *L. fermuntum* S5 were the strongest inducers of IL-10, even higher than the level of the reference *B. longum* CMUL CXL 001 strain, while being weak inducers of IL-12, leading to a high IL-10/IL-12 ratio with similar levels than those obtained for the reference strain *B. longum* CMUL CXL 001.

## DISCUSSION

The human digestive tract is a complex and largely unknown ecosystem. The presence of intestinal microbiota promotes digestion and allows the development of the immune system. The consumption of microorganisms to enhance the function of this microflora or to defense against pathogenic bacteria has given rise to the concept of probiotics. Indeed, the purpose of our study is to select probiotic strains of LAB isolated from baby’s feces for potential application in the health sector as intestinal probiotics. In order to select these strains, they should be subjected to several assessments, such as biofilm formation, antimicrobial effect and recently, immunomodulatory effect. The strains isolated in this study (n=42) were identified by the Vitek^®^ MS technique (MAL-DI-TOF, BioMérieux-France). The MALDI-TOF MS technique, which is based on the identification of a complete protein profile of the bacterium (22), has shown that there is a diversity of intestinal LAB, but the majority of strains have been identified as lactobacilli species (91%) (Fig. 1), and the majority of these strains obtained were identified as *L. rahamnosus/casei/paracasei* (39%) which considered as *Lactobacillus* spp. (Fig. 2).

Generally, probiotic strains could form complex communities, called biofilms, which have several beneficial characteristics for the development and maintenance of a microbial population confronted with different abiotic or biotic factors (23). Maintaining this population required the colonization and preferential adhesion of bacteria to a specific epithelium, such as the intestinal mucosa, extending and stabilizing their residence in the epithelium and excluding pathogenic bacteria by competitive inhibition or by triggering an immune response in the host (24). One of the main characteristics of biofilms is the formation of an Extracellular Polysaccharide (EPS) matrix that helps to self-protection against antibiotics and lytic enzymes, and thus promotes the creation of a microenvironment for metabolic interaction in the population (25). For this purpose, the evaluation of the ability to form a biofilm for our selected strains was measured on 12-well microplates. This type of microplate is hydrophobic, which mimic the conditions of mucous tissues (26). *L. paracasei* isolated from a commercial probiotic supplement was used as reference for a good or moderate biofilm formation. Indeed, the results showed that biofilm formation was strong for 21 strains, moderate for 15 strains and weak for 6 strains. In addition, these 21 strains have a high capacity to form a biofilm, compared with the positive control considered as 100% of this type of capacity (Fig. 3). Moreover, 10 of these strains were *Lactobacillus* spp, and the strain that could form the strongest biofilm was *Lactobacillus* spp S49 (204.95%). Unfortunately, this strongest strain was not identified at the species level by MALDI-TOF technique. In another hand, *L. rhamnosus* S31 showed a capacity of (103.30%) while *L. rhamnosus* S2 (33.05%) could not forma strong biofilm. These results show that a potentially same specie strain such as *L. rhamnosuscan* give different capacities to form a biofilm. This confirms the hypothesis that biofilm formation is strain-dependent as described before (27). Indeed, the metabolic pathways that trigger the formation of biofilm, and the adhesion factors involved, depend on the organism concerned and the environment in which it forms thenbiofilm (28).

Commensal microorganisms prevent pathogenic colonization phenomena through competitive processes: nutrient metabolism, pH modification, secretion of antimicrobial peptides, effects on cell signaling pathways (limitation of virulence factors). The antibacterial effects induced by the microbiota improve the host’s response to pathogens. The effect studied in our case is the production of thermoresistant antimicrobial substances which are potentially bacteriocins or Bacteriocin-Like Inhibitory Substances (BLIS) to control the 2 pathogens used as a model of Gram-positive (*L. monocytogenes* ATCC^®^ 19115™) and Gram negative (Enteropathogenic *E. coli* ATCC^®^ 8739™) bacteria. *L. monocytogenes* ATCC^®^ 19115™ was used as a susceptible strain to bacteriocins. In order to select the strains with a potential antimicrobial effect, the heat-treated supernatant from a 24-hour culture of each strain was added in the presence of the pathogens already mentioned, and then the growth of each pathogen in the presence of the supernatant was evaluated by measuring the OD every 2 hours for 18 hours. Supernatants have been heat treated to degrade all other thermolabile antibacterial substances. The strain with a supernatant that inhibits pathogen growth was considered a strain that produces a heat-resistant antibacterial substance. Furthermore, the *E. faecium* CMUL20-2 (17) strain was used as a positive control for *L. monocytogenes* since it showed an anti-*Listeria* activity as shown by Al Kassaa et al. (17). Overall, most of the isolated LABs showed a remarkable inhibitory effect against one or both pathogenic strains, which is similar to another study that demonstrated the significant antimicrobial activity of the LAB against Gram positive and Gram-negative pathogenic strains (29). However, LAB strains can produce inhibitory substances different from bacteriocins and therefore, more tests should be conducted in order to confirm the presence of secreted bacteriocins such as the protease treatment test, extraction method and LCMS for purification as well as molecular weight determination (30, 31).

The intestinal microbiota plays an essential role in the development and maturation of the immune system, and therefore on its functions. Some bacteria stimulate particularly pro-Th1 responses in intestinal mucosal immune system which promotes the production of pro-inflammatory cytokines such as IL-12, while other microbial strains stimulate regulatory lymphocytes T (Treg) by promoting the production of anti-inflammatory cytokines such as IL-10 (32). The composition of the microbiota therefore plays a major role in the balance between pro- and anti-inflammatory immune responses, which is essential for maintaining intestinal homeostasis (32). Hence, the immunomodulatory capacities of isolated strains were studied after stimulation of PBMCs using heparinized blood from five healthy donors. The different strains were brought into contact with the isolated PBMCs, in cell culture wells, from each donor independently for 24 hours. Then, the cell culture supernatant was analyzed for the concentration of IL-10 and IL-12p70 secreted by PBMCs in the presence of each selected strain. *B. longum* CMUL CXL 001 was used as positive control for IL-10 PBMCs stimulation since this strain was showed this anti-inflammatory capacity as mentioned by Zaylaa et al. (21). In another hand, *E. mundtii* IAK, was shown to stimulate the pro-inflammatory response in PBMCs model (unpublished data). The results were evaluated by calculating means of the values from the five donors with a standard deviation. 14 strains stimulated IL-10 production with values above 1000 pg/ml. *L. rhamnosus* S8P was the best IL-10 producing strain (2670, 539 pg/ml). On the other hand, 3 strains stimulated the production of pro-inflammatory interleukin “IL-12p70” more than 50 pg/ml. *Leuconostoc lactis* S69 was the best producing strain of IL-12p70 (108.53 pg/ml). The ratio (IL-10/IL-12p70) was not calculated for all strains because the production of the IL-12 was very low compared to the production of IL-10. Hence, all our strains are able to stimulate an anti-inflammatory response with different stimulation levels. Indeed, previous studies have shown that probiotic strains of intestinal origin have an anti-inflammatory effect (33) which is confirmed by our study where all isolated strains are anti-inflammatory strains.

Of all isolated strains, 7 strains (*L. rhamnosus* S8G, *Lactobacillus* spp. S14, *L. paracasei* S23, *Lactobacillus* spp. S34, *Lactobacillus* spp. S36, *Lactobacillus* spp. S49, *Lactobacillus* spp. S67) have shown a strong immunobiotic/probiotic effect based on the characteristics already mentioned, showing a strong ability to form a biofilm, antibacterial activity against the 2 pathogenic strains used, as well as having the anti-inflammatory ability by stimulating a high rate of IL-10.

## CONCLUSION

In this study, several LAB strains of fecal origin from breast-feeding babies, were selected for their potential probiotic and immunobiotic effect. Based on the probiotic properties studied, two best probiotic strains with anti-inflammatory effects can be selected: *Lactobacillus* spp. S14 and *Lactobacillus* spp. S49. These two strains have important biofilm formation capacities, marked antibacterial effects against *L. monocytogenes* and *E. coli* via a heat-resistant molecule as well as an important immunomodulatory effect as they were able to stimulate high IL-10 production.

In future studies, these two strains will be a subject for *in vivo* application and commercialization as probiotics with anti-inflammatory effect. Eventually, these two strains may be used by patients with chronic diseases such as IBD (e.g. Crohn’s diseases), Familial Mediterranean Fever (FMF) disease and even other diseases caused by low-grade inflammation. In this context, further characterization should be performed on these two strains, such as stability in host, strain encapsulation and other pharmacological characteristics.
